# Implementation of a prehabilitation program before abdominal wall surgery: a pilot and feasibility study

**DOI:** 10.1007/s10029-025-03325-8

**Published:** 2025-04-08

**Authors:** Gaëtan-Romain Joliat, Sonia Krouk, Eddy Cotte, Guillaume Passot

**Affiliations:** 1 Department of General Surgery and Surgical Oncology, Centre Hospitalier Universitaire Lyon Sud, 165, chemin du Grand Revoyet, Pierre-Bénite, 69495 France; 2 Department of Visceral Surgery, Lausanne University Hospital CHUV, University of Lausanne (UNIL), Lausanne, Switzerland

**Keywords:** Optimization, Physical activity, Nutrition, Hernia, Preoperative conditioning

## Abstract

**Purpose:**

Prehabilitation in abdominal wall surgery (AWS) might improve postoperative outcomes, but current data are scant. A prehabilitation program before AWS, including specific hypopressive abdominal exercises, was recently implemented in our department. This study aimed to present the characteristics of the implemented program and to assess the adherence rate to hypopressive abdominal exercises.

**Methods:**

A retrospective study of all consecutive patients included in the pathway from October 2021 to October 2024 was performed. The multimodal prehabilitation program included nutritional support, physical activities (cardiorespiratory training, muscular strengthening, hypopressive abdominal exercises, and relaxation), and psychological support. Adherence rate was defined as the number of patients who performed the proposed abdominal exercises divided by the total number of included patients.

**Results:**

A total of 103 patients were included (43% women, median age: 64, IQR 55–72, median body-mass index: 29 kg/m^2^, IQR 26–33). Most of them had a midline hernia (*n* = 79, 77%) and underwent a retromuscular mesh repair (*n* = 93, 90%). Ninety-six patients were adherent to the hypopressive abdominal exercises (adherence rate: 93%). Obese patients had a significantly lower adherence rate to hypopressive abdominal exercises than non-obese patients (29/34 = 85% vs. 67/69 = 97%, *p* = 0.025). Median length of hospital stay was 3 days (IQR 2–5) and postoperative complications occurred in 29 patients (28%).

**Conclusion:**

The implementation of a prehabilitation program in AWS was feasible. Moreover, adherence to the hypopressive abdominal exercises was high. Obese patients might require more attention to improve their adherence to the program.

## Background

Prehabilitation was developed over the past years to optimize patients before surgery [1–3]. This concept was recently described as a preoperative multimodal conditioning of patients with the aim of decreasing postoperative morbidity and improving recovery after surgery. Interventions such as nutritional support, physical activities, or psychological support were proposed. Several studies have shown that prehabilitation improved postoperative outcomes (decrease of length of hospital stay and postoperative complications) after major abdominal surgery, especially for multimorbid and frail patients [3–7]. Prehabilitation in abdominal wall surgery (AWS) remains nevertheless controversial with a scarcity of data published in the current literature [8]. Even with the development of minimally-invasive techniques, AWS still induces significant postoperative complications, in particular wound-related morbidity (surgical-site occurrences) [9]. Moreover, patients presenting with complex or recurrent hernias often have comorbidities (e.g., obesity, diabetes mellitus, or respiratory pathologies) and past surgical history. Their functional capacity and physiologic reserve can consequently be diminished. These patients might therefore benefit from a well-conducted prehabilitation program.

In addition, elements that need to be included in a prehabilitation program also remain presently unknown. It seems important to improve and act on modifiable risk factors of complications, such as smoking or obesity and to have the patients in their best physical and nutritional shape to face an abdominal wall operation [8].

In this context, a multimodal prehabilitation program in AWS was implemented in our unit to optimize patients more at risk of complications and patients undergoing complex abdominal wall reconstruction operations.

The aim of the current study was to present the multidisciplinary and multimodal prehabilitation pathway that was implemented and to evaluate the adherence to the preoperative hypopressive exercise program.

## Methods

### Patients and eligibility criteria

Prehabilitation for major abdominal surgery was implemented in February 2018 in the Department of General Surgery and Surgical Oncology of Lyon Sud Hospital, Lyon, France. A specific prehabilitation for AWS patients started in October 2021.

All consecutive patients selected for a prehabilitation before AWS were included in this study. Data of these patients were prospectively collected. Patients with giant hernias (hernia defect > 10 cm) and loss of domain [10], with a body-mass index (BMI) > 25 kg/m^2^, with malnutrition (nutritional risk screening ≥ 3) 11], or with significant cardiac, respiratory or metabolic (such as diabetes mellitus) comorbidities were included. Patients with contraindication for abdominal exercises, age <18, or refusal to participate in the program were excluded from the study.

### Endpoints

The primary endpoint of the study was the rate of patients who were adherent to the program of hypopressive abdominal exercises (i.e., the number of patients who actually performed the hypopressive abdominal exercises divided by the number of included patients).

Other endpoints were the number of patients who did the physical exercises at home and in the outpatient clinic with our physical activity specialist, the program feasibility (number of patients who completed the program), the 90-day complication rate, and the length of hospital stay. Complications were defined based on the Dindo-Clavien classification [12]. Major complications were defined as grade ≥3a.

### Prehabilitation program

**Fig. 1:**
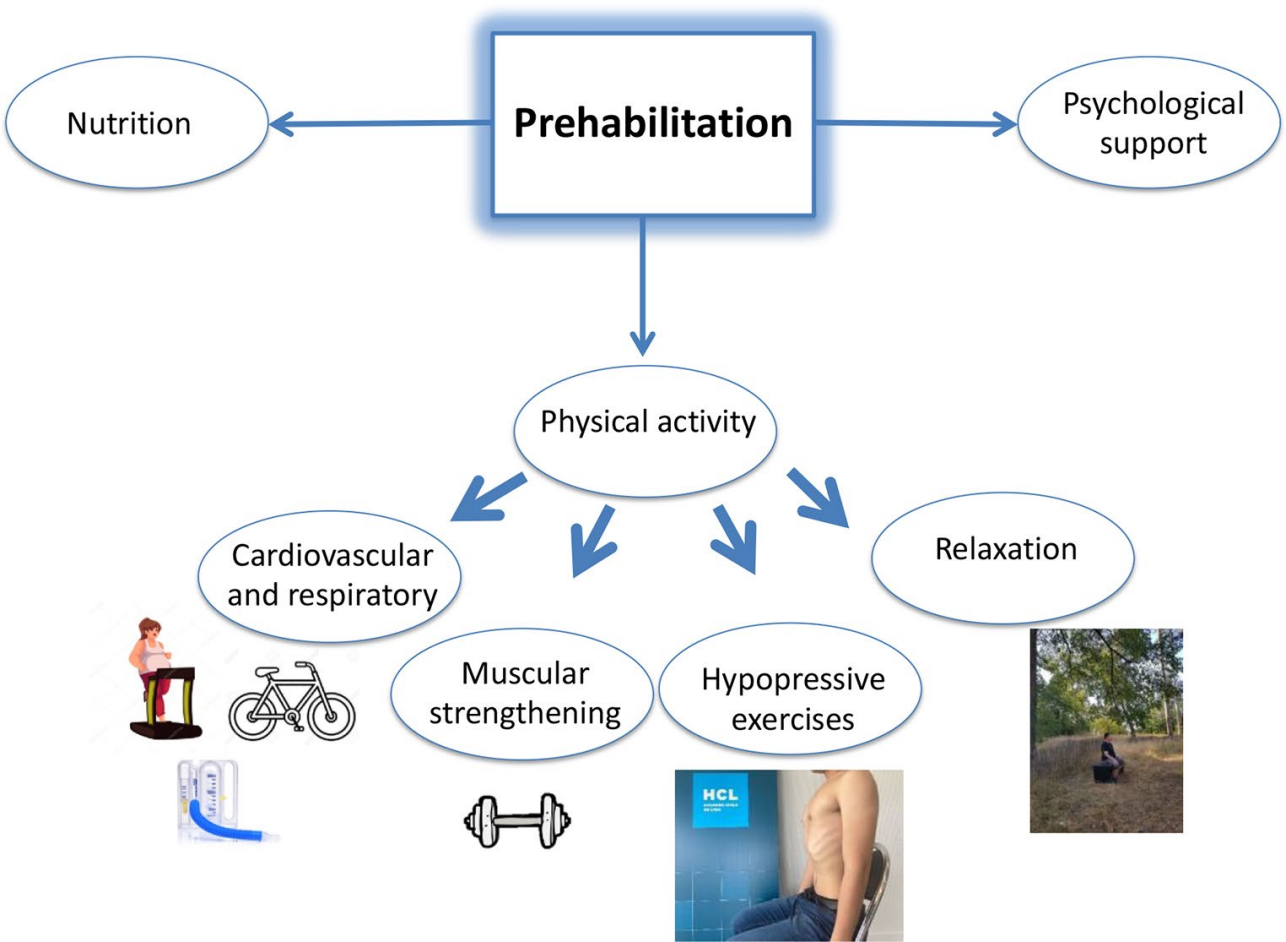
Summary of the implemented prehabilitation pathway for abdominal wall surgery

In the unit, a multimodal prehabilitation program including nutritional support, psychological support (including smoking cessation consultation), and physical activity was implemented. General characteristics of the program are depicted in a previous article [13] and can be found on the dedicated website of our unit (https://www.chu-lyon.fr/prehabilitation-avant-chirurgie-majeure). Figure 1 summarizes the prehabilitation pathway.

Regarding nutrition, all patients were assessed regarding their BMI, weight loss/gain, and daily calory intake. In case of malnutrition or overweight/obesity, patients were referred to a nutritionist. All patients received diet advice, and oral nutritional supplements were given. Malnutrition screening and nutritional support were based on the SFAR (Société Française d’Anesthésie et de Réanimation, French Society of Anesthesia and Resuscitation) and SFNEP (Société Francophone de Nutrition Entérale et Parentérale, Francophone Society of Clinical Nutrition and Metabolism) recommendations [14].

Psychological support was performed by a weekly call with patients. The aim of the calls was to reduce patient anxiety (using potential clarifications of the program or explanations of the surgical journey), to offer patients a regular contact person, and to improve adherence to the prehabilitation pathway (positive reinforcement). For smokers, it was explained that their risk of wound problems postoperatively was importantly increased by tobacco consumption. They were required to stop smoking before the operation. An advice booklet was given and a consultation with an addiction specialist was scheduled if needed.

**Fig. 2:**
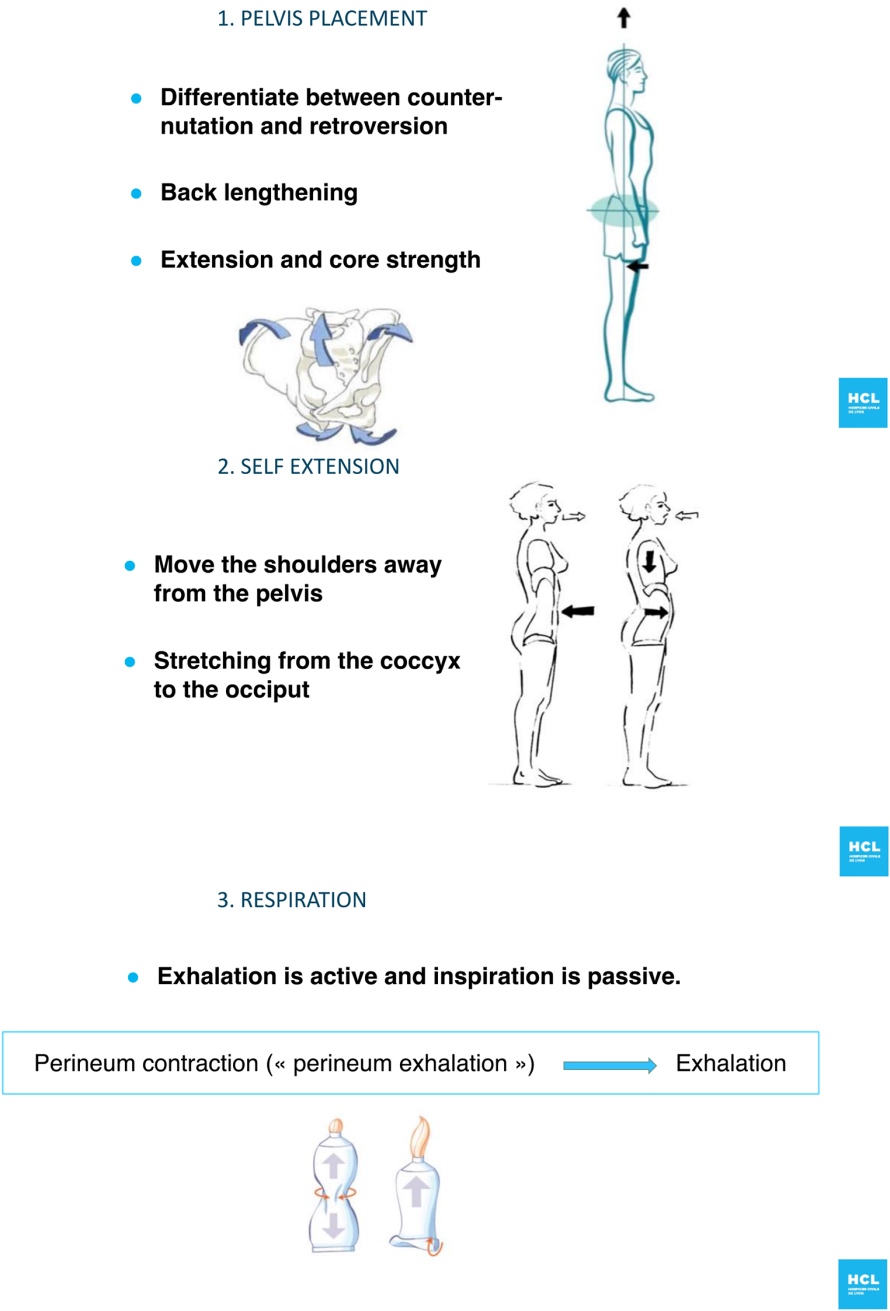
Illustration of one hypopressive abdominal exercise included in the prehabilitation program. A complete video by our sport coach explaining how to perform hypopressive abdominal exercises can be found on https://www.youtube.com/watch?v=sbogqyeDvfk

Physical activity was promoted using cardiovascular and respiratory exercises (endurance), muscular strengthening, relaxation (breathing exercise, meditation, cardiac coherence), and hypopressive abdominal exercises. Once the patient was included in the prehabilitation program, a personal interview with the physical activity instructor was performed. Patient abilities and baseline performances were assessed using 3 simple physical tests: 6-minute walk, hand grip strength, and sit-to-stand. The 6-minute walk is a test measuring the distance a patient can cover during 6 min of walking. The hand grip strength was measured using a hand dynamometer. The highest value between both hands was recorded. The sit-to-stand test measures the number of times the patient can stand up from a seated position in a chair within 30 s. Based on the interview and the results of the tests, a tailored program including cardiovascular exercises, muscular strengthening, relaxation, and hypopressive abdominal exercises was proposed to the patient. Endurance activities (such as walking, jogging, cycling, or swimming minimum 3 sessions per week) were recommended at a moderate intensity based on the Borg scale [15]. Incentive spirometry was proposed as breathing exercises. Relaxation was defined as exercises helping the recovery after training and to decrease the stress level. Relaxation exercises advised to patients were breathing exercises, meditation sessions, and cardiac coherence exercises. Hypopressive abdominal exercises are physiological movements based on biomechanics and body posture. Moreover, hypopressive abdominal exercises could be particularly well suited for patients with ventral hernia by strengthening the abdominal muscles (especially the transverse abdominis and the internal oblique muscle), reinforcing the back position, and avoiding high rise of abdominal pressure [16]. Hypopressive abdominal exercises are based on the pelvis placement in line with the perineum, the stretching from the coccyx to the occiput (self extension), and active exhalation with perineum contraction. The main important movement of hypopressive abdominal exercises is to contract the perineal region during breathing out. This induces the abdominal wall to sink in. These exercises can be done seated, standing, or supine. A video made by the sport coach of our department on how to perform these exercises is freely available on internet (https://www.youtube.com/watch?v=sbogqyeDvfk). An hypopressive abdominal exercise is illustrated in Fig. 2. Hypopressive exercises were recommended to be performed 3 to 5 times a week during 20-minute sessions. Intensity of these exercises were dependent on the basal physical condition of the patients. Among patients who felt the exercises were too easy, it was recommended to increase the frequency and duration of the exercises.

Minimum length of the prehabilitation period was 4 weeks. If that was not possible because of severe symptoms or logistic issues requiring surgery more rapidly, the program was adapted specifically. Postoperatively, patients followed an enhanced recovery after surgery program.

### Statistics and ethics

Descriptive statistics were used to summarize the data. Continuous data were presented using median and interquartile range (IQR) and categorical data using number and percentage. All statistical calculations were done with SPSS 29.0 (IBM Corp., Armonk, NY, USA).

This study was granted approval by the local institutional review board and was realized in accordance with the Declaration of Helsinki.

## Results

### Patients and surgical details

A total of 103 patients were included during the study period. Basic demographics and preoperative characteristics are summarized in Table 1. The patient cohort was composed of a majority of men (57%). Median age of the entire cohort (*n* = 103) was 64 (IQR 55–72) and median BMI was 29 kg/m^2^ (IQR 26–33). The surgical characteristics are shown in Table 2. The median time between initial consultation and operation was 105 days (IQR 77–122). No emergency hernia surgery was necessary during the prehabilitation in the entire cohort.

**Table 1:** Demographics and preoperative characteristics of included patients (*n* = 103)

	Median or number	IQR or percentage
Sex (women/men)	59/44	43%/57%
Age, years	64	55–72
Weight, kg	84	73–94
Height, cm	170	160–175
BMI, kg/m^2^	29	26–33
Waist size, cm	109	100–117
ASA score I/II	73	71%
Active smoking	16	16%
Diabetes mellitus	20	19%

BMI: body-mass index, ASA: American Society of Anesthesiologists, IQR: interquartile range

**Table 2:** Surgical and operative characteristics of included patients (*n* = 103)

	Median or number	IQR or percentage
Botulinum toxin injection	62	60%
Operation duration, min	120	120–180
Hernia location		
Midline Lateral Parastomal Inguinal	79 16 6 2	77% 16% 6% 1%
Complex hernia^+^	80	78%
Hernia neck size (width), cm	10	6–12
Open surgery	82	80%
Mesh placement*		
Onlay Sublay Bridge	2 93 2	2% 90% 2%
Component separation		
Posterior (TAR) Anterior (Ramirez)	33 30 3	32% 29% 3%

^+^ Complex hernias were defined as hernias > 10 cm, hernias with > 20% of loss of domain, parastomal hernias, lumbar hernias, lateral hernias, or subcostal hernias

* No mesh was placed in 6 cases due to a contaminated surgical field

TAR: transverse abdominis release, IQR: interquartile range

### Preoperative evaluation

Regarding initial evaluations before starting the prehabilitation program, the median distance covered in 6 min (6-minute test) was 525 m (IQR 475–574). Median handgrip strength was 34.9 kg (IQR 26.3–46.2) in the strongest arm. The median number of repetitions for the sit-to-stand test was 20 (IQR 16–24). Regarding the 6-minute walk test, patients had a median improvement of 47 m (IQR 20–55, mean improvement 44 ± 28 m) after prehabilitation. For the hand grip strength, patients increased their hand strength by a median of 2 kg (IQR 0–4, mean 2.3 ± 3.5 kg) after prehabilitation. The median number of sit-to-stand within 30 s was increased by 2 (IQR 1–4, mean increase 2.9 ± 3.5).

After prehabilitation, patients lost a mean of 3 ± 4 kg (median loss 2 kg, IQR 0–5). In the subgroup of obese patients (BMI > 30 kg/m^2^, *n* = 34), the mean weight loss after prehabilitation was 4 ± 5 kg (median loss 3 kg, IQR 1–6). Among the 43 patients who smoked before starting the prehabilitation program, 27 patients quitted smoking after the program before the operation.

### Physical activities and adherence to hypopressive abdominal exercises

Among the 103 included patients, 16 patients (15%) did the full exercise program with the hospital physical activity specialist during outpatient sessions, 85 (83%) did it at home (using the provided videos or with the help of a physiotherapist), and 2 (2%) trained in the hospital and at home.

**Fig. 3:**
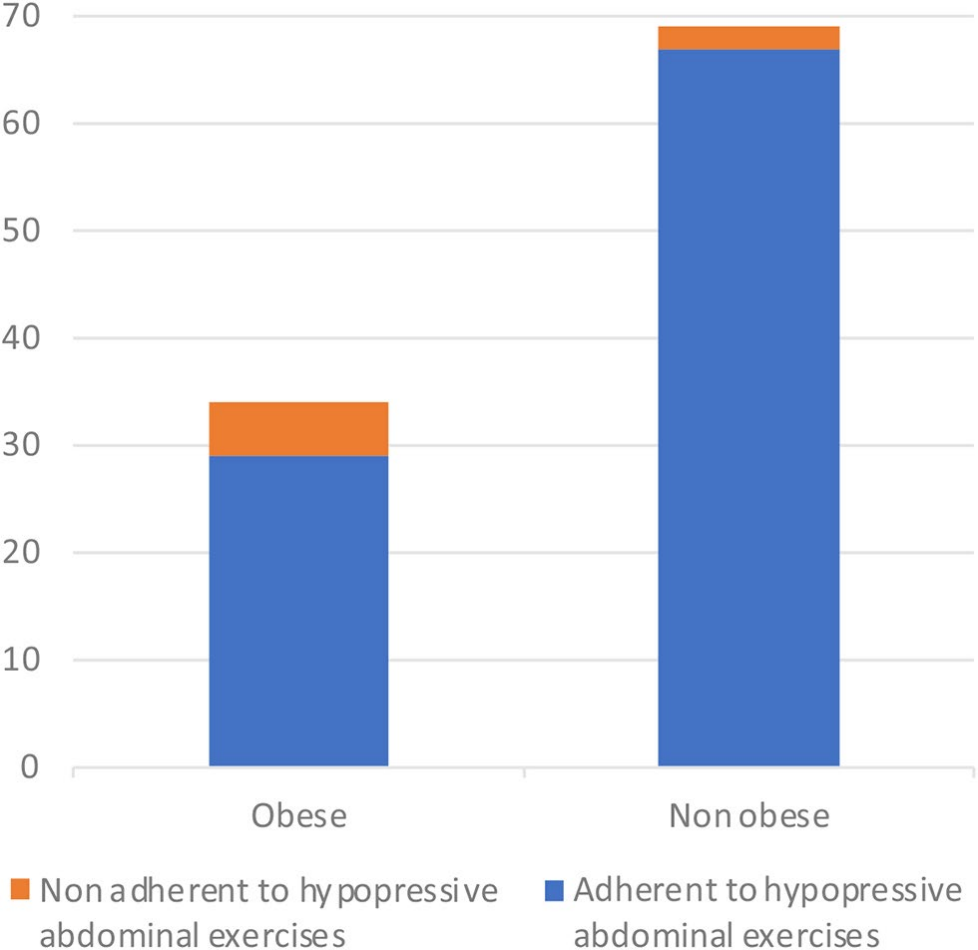
Bar plots of obese and non obese patients adherent to the hypopressive abdominal exercises (29/34 = 85% vs. 67/69 = 97%, *p* = 0.025)

Ninety-six patients were adherent to the hypopressive abdominal exercises (adherence rate: 93%=96/103). The adherence rate to the hypopressive abdominal exercises was similar between elderly (age > 70, *n* = 27) and younger patients (26/27 = 96% vs. 70/76 = 92%, *p* = 0.457). On the contrary, obesity (median BMI > 30 kg/m^2^, *n* = 34) did have a negative effect on the adherence rate to hypopressive abdominal exercises (adherence rates among obese and non-obese patients: 29/34 = 85% vs. 67/69 = 97%, *p* = 0.025, Fig. 3).

### Postoperative outcomes

Median length of hospital stay was 3 days (IQR 2–5). Postoperative complications occurred in 29 patients (overall morbidity rate: 28%). Major complications (Dindo-Clavien ≥3a) occurred in 13 patients (13%). Eight patients had to be reoperated on (8%). Surgical-site occurrences developed in 20 patients (19%). Two patients (2%) presented a recurrence during the study period.

## Discussion

This pilot study on prehabilitation before AWS showed that the implemented program was feasible with 103 included patients and had a high adherence rate regarding hypopressive abdominal exercises (93%).

The adherence to the hypopressive abdominal exercises was high in the entire cohort (96/103). This can potentially be explained in parts by the support that the patients received [17]. Patients had a first interview with the physical activity specialist before starting the prehabilitation. This permitted to give all information regarding the prehabilitation to the patients and for the patient to have an identified contact person responsible of the program. Moreover, patients were contacted every week by the physical activity specialist. This helped to motivate the patients and to answer all potential remaining questions. Finally, information booklets and exercise videos were also provided to the patients to accompany them during their preoperative pathway. Interestingly, adherence rate was lower in obese patients than in non-obese patients (29/34 vs. 67/69, *p* = 0.025). Obese patients might potentially need to have a tighter follow-up or to be accompanied in person to ensure that the preoperative hypopressive abdominal exercises are performed.

Hypopressive abdominal exercises are not new, but their application in prehabilitation for AWS is original and innovative. The use of hypopressive abdominal exercises might be particularly adapted to patients with ventral hernia as it reinforces the abdominal wall with less pressure and applied tension on the muscles than hyperpressive exercises [16, 18]. Furthermore, patients can continue these exercises during the postoperative period as no abrupt abdominal movements are required. Data on hypopressive abdominal exercises are currently scarce, and no data exist regarding their use in a perioperative context. In a recent systematic review published in 2024, the authors found that heterogeneity regarding execution, follow-up, and standardization of hypopressive gymnastics was high among all included articles [16, 18]. These divergences in methods should be taken into account in the result interpretation.

The preoperative optimization of patients due to prehabilitation has the objective of preparing the patients to face AWS in their best physical condition. The mechanisms of potential improved postoperative outcomes after prehabilitation are mainly based on the management of existing comorbidities. Regulation of blood sugar in diabetes patients, weight loss in overweight or obese patients, smoking cessation, or nutritional support in malnourished permit to improve postoperative morbidity. Moreover, strengthening the abdominal wall muscles might contribute to improve the strength of the repair and patient reported outcomes but these hypotheses remain to be proven.

Several limitations of the study need to be mentioned. The retrospective design could have induced errors during data collection. Moreover, missing data can also bias the results. A control group of patients without prehabilitation was not available precluding any comparisons. It was therefore not possible to evaluate the potential benefits of prehabilitation, but the aim of the present study was to focus on the feasibility and adherence to the prehabilitation program.

This study paves the way for a future comparison study assessing the outcomes after AWS of patients with and without prehabilitation to evaluate the potential clinical benefits of prehabilitation. Regarding clinical perspective, the next step would be to tailor even more specifically the prehabilitation program to individual patients based for example on patient comorbidities or on bioelectrical impedance analyses.

In conclusion, this pilot study showed that prehabilitation before AWS was feasible with a high adherence rate. Implementation of such a preoperative pathway requires multidisciplinary collaboration, coordinated organization, and detailed planning to obtain good results.
